# Supplementary material for: Hyperbranched Polycaprolactone through RAFT Polymerization of 2-Methylene-1,3-dioxepane
Source: Polymers (Basel). 2019 Feb 13;11(2):318. doi: 10.3390/polym11020318 (PMC6419385; doi:10.3390/polym11020318)
Supplement: Supplementary file 1 [file polymers-11-00318-s001.pdf]

Hyperbranched Polycaprolactone through RAFT Polymerization of 2-Methylene-1, 3-dioxepane

Ping Xu[1], Xiaofei Huang[1,2], Xiangqiang Pan[1], Na Li[1*], Jian Zhu[1*], and Xiulin Zhu[1, 3]

[1] State and Local Joint Engineering Laboratory for Novel Functional Polymeric Materials, Jiangsu Key Laboratory of Advanced Functional Polymer Design and Application, Department of Polymer Science and Engineering, College of Chemistry, Chemical Engineering and Materials Science, Soochow University, Suzhou 215123, China.

[2] Jiangsu Litian Technology Co. Ltd., Rudong County, Jiangsu 226407, China

[3] Global Institute of Software Technology, No 5. Qingshan Road, Suzhou National Hi-Tech District, Suzhou 215163, China.

* Correspondence: Na Li: chemlina@suda.edu.cn; Jian Zhu: chemzhujian@suda.edu.cn; Tel.: +86-512-65880726

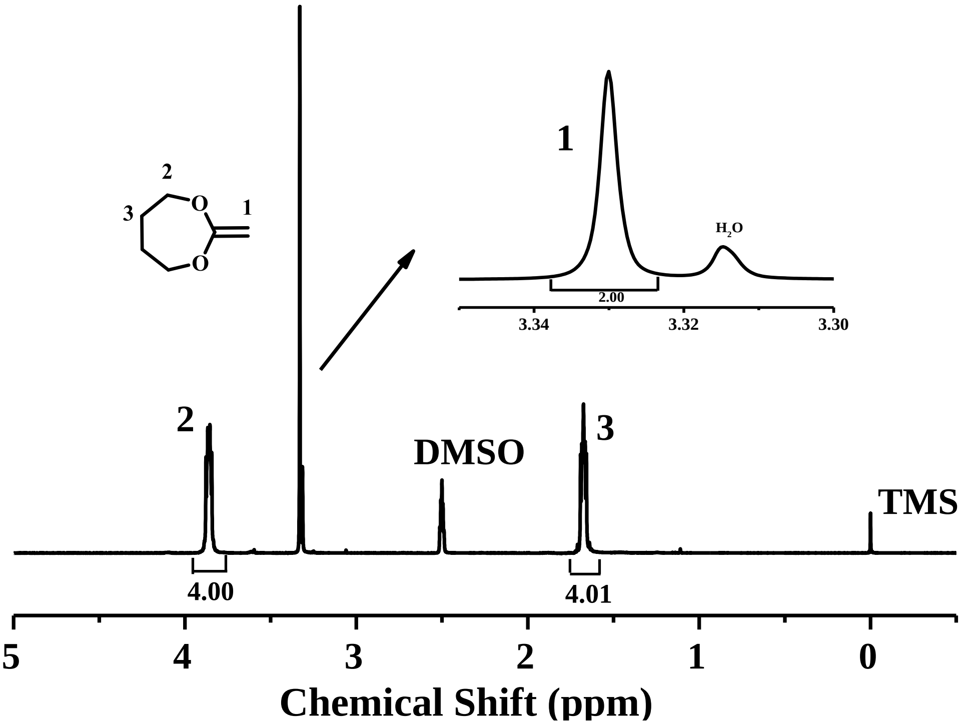

Fig. S1 ^{1}H NMR spectrum of MDO.

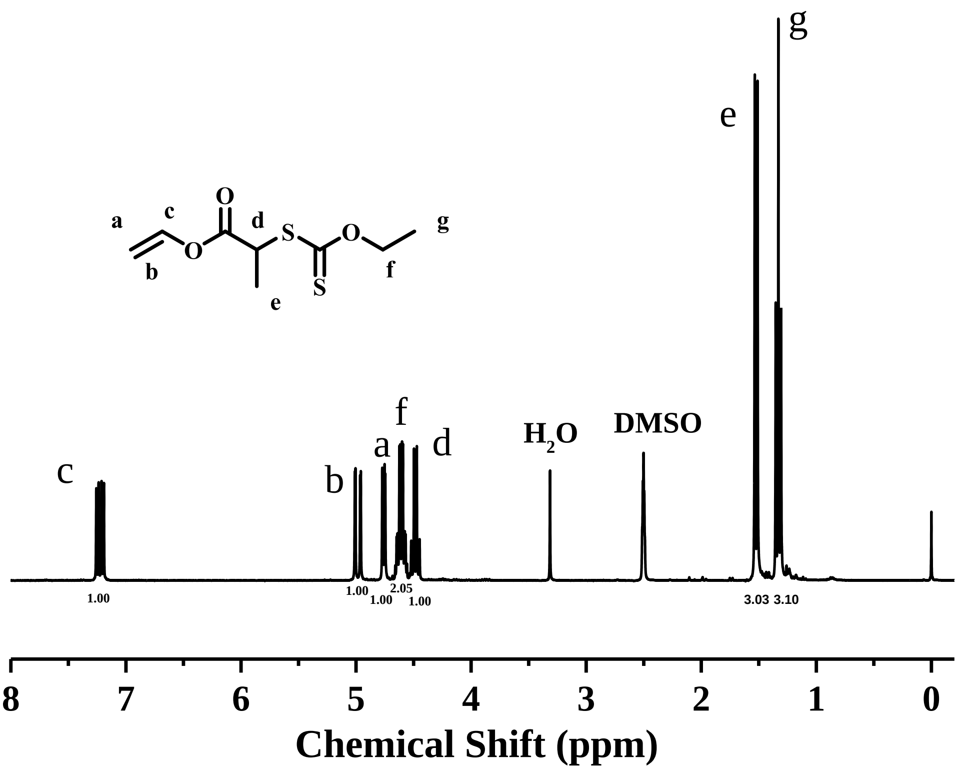

Fig. S2 ^{1}H NMR spectrum of ECTVP.

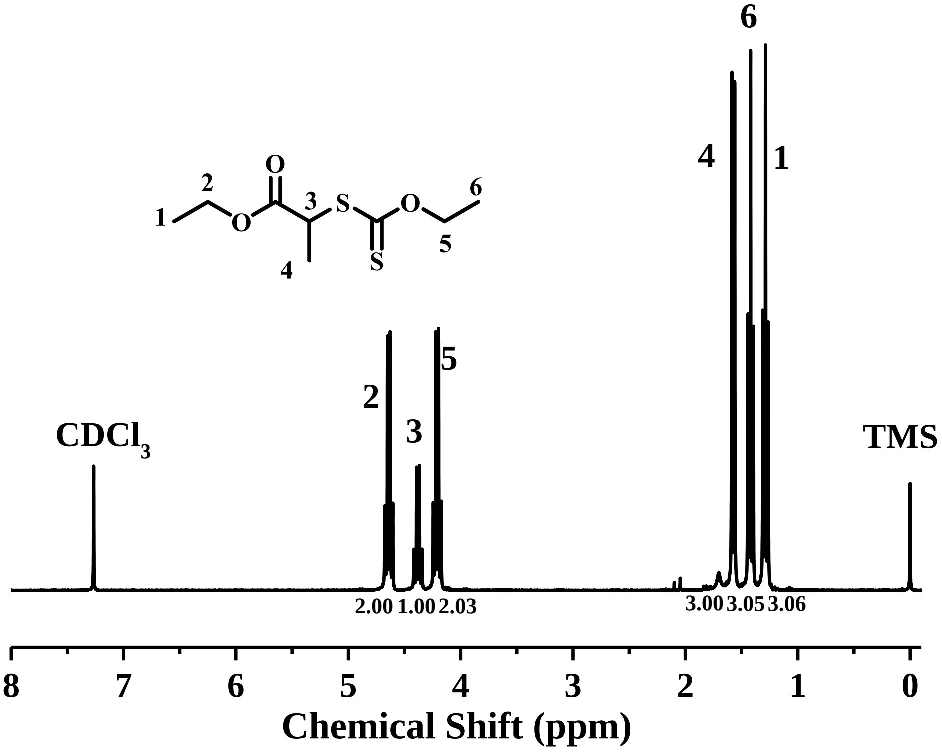

Fig. S3 ^{1}H NMR spectrum of EXEP.

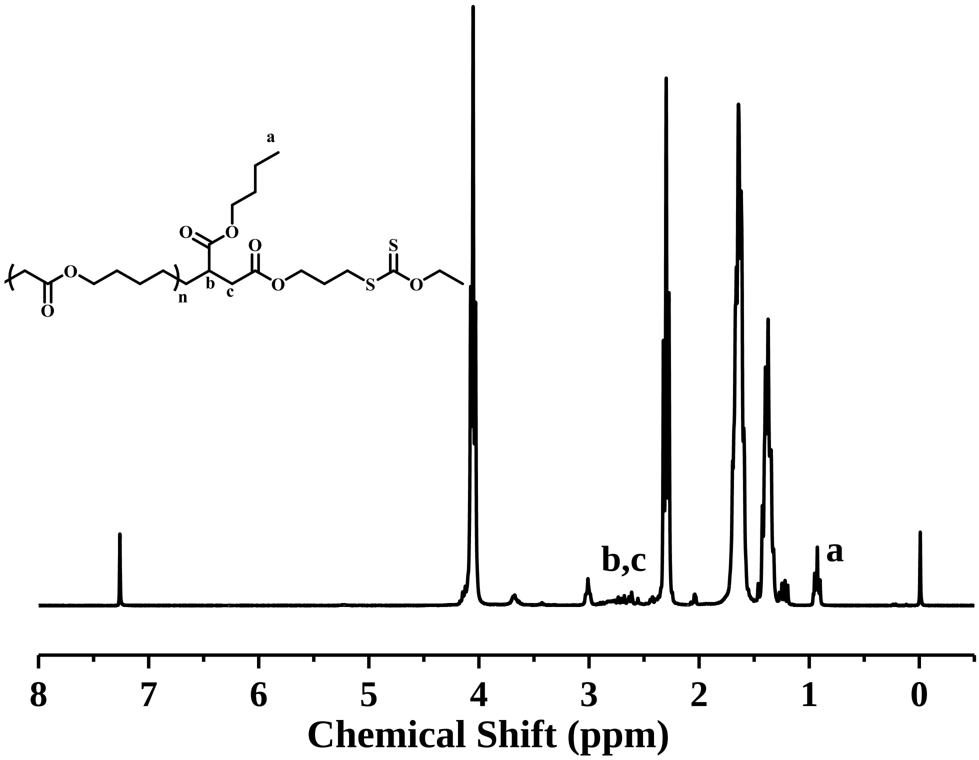

Fig. S4 ^{1}H NMR spectrum of linear PCL in Table 1 Entry P5.

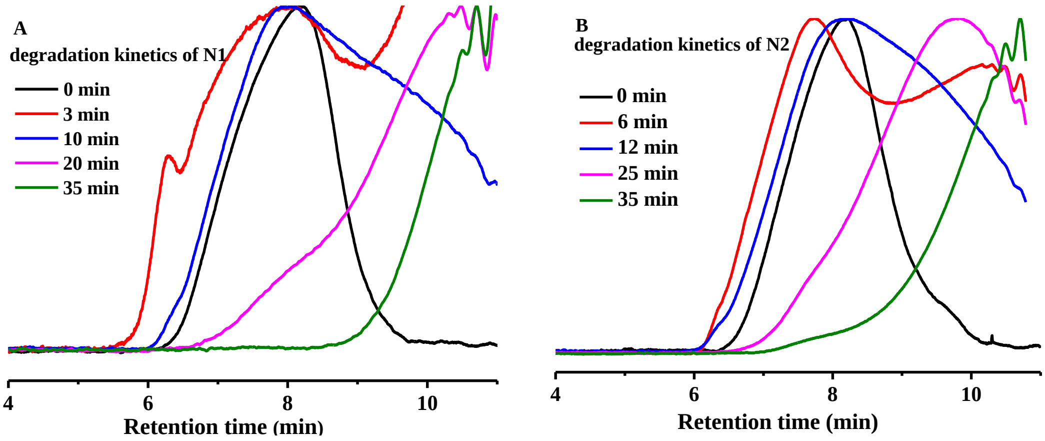

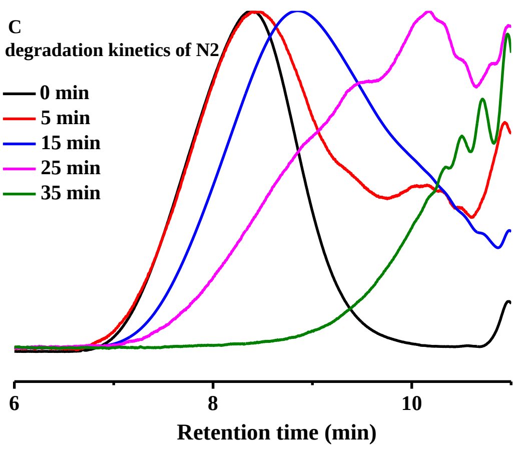

Fig. S5 GPC trace of the hydrolysis of hyperbranched polymers N1(A), N2 (B) and linear polymer N3(C) for different time points in a solution of KOH in methanol (0.025 M) at 40 °C.
